# Larvicidal, Ovicidal, and Repellent Activities of *Leucas stachydiformis* (Hochst. ex Benth.) Briq Essential Oil against *Anopheles arabiensis*

**DOI:** 10.1155/2024/1051086

**Published:** 2024-03-29

**Authors:** Sisay Fikru, Ketema Tolossa, Peter Lindemann, Franz Bucar, Kaleab Asres

**Affiliations:** ^1^Department of Pharmaceutical Chemistry and Pharmacognosy, School of Pharmacy, College of Health Sciences, Addis Ababa University, P.O. Box 1176, Addis Ababa, Ethiopia; ^2^Endod and Other Medicinal Plants Research Unit, Aklilu Lemma Institute of Pathobiology (ALIPB), Addis Ababa University, P.O. Box 1176, Addis Ababa, Ethiopia; ^3^Institut für Pharmazie, Martin Luther Universität Halle Wittenberg, Hoher Weg 8, Halle D-06120, Germany; ^4^Department of Pharmacognosy, Institute of Pharmaceutical Sciences, University of Graz, Graz, Austria

## Abstract

Larvicidal, ovicidal, and repellent activities of the essential oil extracted by hydrodistillation from the leaves of the endemic Ethiopian plant *Leucas stachydiformis* (Hochst. ex Benth.) Briq were investigated against *Anopheles arabiensis*, the dominant malaria vector species in Ethiopia with the objective of searching for a plant-based malaria vector control strategy from medicinal plants. The larvicidal effect was tested against the fourth instar *An. arabiensis* wild larvae whilst freshly laid ova of *An*. *arabiensis* were used to determine the ovicidal activity of the essential oil at concentrations ranging from 6.25 to 400 ppm. Concentrations of 41.6–366.7 *µ*g/cm^2^ were used to evaluate the repellent activity of the essential oil on 3–5 days old adult female *An. arabiensis*. The oil composition of *L. stachydiformis* was also analyzed using GC-MS. The study revealed that the oil possesses the highest larvicidal activity at 400 ppm and 200 ppm after 24 h and 48 h of treatment. LC_50_ values for the fourth larval instar after 24 h and 48 h of treatment were 43.4 ppm and 34.2 ppm, respectively. After 72 h of exposure, the oil displayed 100% ovicidal activity at 400 ppm with an IH_50_ value of 32.2 ppm. In the repellency test, at concentrations of 366.7, 133.3, and 41.6 µg/cm^2^, the oil gave a total percentage protection of 67.9 ± 4.2%, 37.2 ± 2.8%, and 32 ± 2.2%, respectively, for 4 h. The highest concentration (366.7 *µ*g/cm^2^) gave 100% protection up to 90 min. GC-MS analysis of the oil revealed the presence of 24 compounds representing 90.34% of the total oil with caryophyllene oxide, germacrene D, and *trans*-caryophyllene constituting more than 50% of its components. Results of the present study suggest that the essential oil of *L*. *stachydiformis* has the potential to be used for the control of *An. arabiensis* mosquitoes.

## 1. Introduction

Despite tremendous efforts made to curb malaria morbidity and mortality, further progress in malaria control has slowed down recently [1]. According to the latest WHO report, globally, there were 247 million cases of malaria in 2021 compared to 245 million cases in 2020 [2]. Vector control is one of the prevention tools to mitigate mosquito-borne diseases including malaria [3]. In this regard, community-based long-lasting insecticidal nets (LLINs) and larval source management (LSM) have been the mainstay in vector control efforts [4]. However, these synthetic insecticide-based methods are threatened because of widespread resistance to all currently used insecticides by mosquitoes, and their negative effect on the nontarget organisms and the ecosystem [5]. These problems may lead to renewed malaria outbreaks and deaths from insecticidal poisoning. Above all, the situation is worsening in developing countries as the control of malaria vectors predominantly relies on these insecticide-based methods and due to the improper use of personal protection [6, 7].

Thus, there is an urgent need to search and develop new malaria vector control methods which are more environmentally safe, biodegradable, and target-specific insecticides [8]. Identification of potential insecticides and repellents from natural product resources, such as plant extract and essential oils is one solution for the abovementioned problems. Previous studies have demonstrated that members of the genus *Leucas* possess larvicidal [9–12], ovicidal [13], and repellent [14] activities. Therefore, the present study was designed to investigate the larvicidal, ovicidal, and repellent activities of the essential oil of leaves of *Leucas stachydiformis* (Hochst. ex Benth.) Briq against *An. arabiensis*, the dominant malaria vector in Ethiopia.

## 2. Methods

### 2.1. Plant Material and Extraction of Essential Oil

The leaves of *L. stachydiformis* were collected in August 2022 from Gulelle Botanical Garden (GBG), Northwest of the capital Addis Ababa, Ethiopia, located at 9°08′39″N, 38°44′98″E and 2770 m altitude above sea level. The authenticity of the plant material was confirmed by Dr. Birhanu Belay of GBG and a voucher specimen with the collection number SF001/2021 was deposited at the National Herbarium, Department of Plant Biology and Biodiversity Management, College of Natural Sciences, Addis Ababa University, Addis Ababa, Ethiopia. The essential oil was extracted by hydrodistillation using a Clevenger-type apparatus. The residual water from the oil was removed by anhydrous sodium sulfate, Na_2_SO_4_ (CAS no. 7757-82-6), and the oil was stored in a refrigerator in a tightly closed amber-colored bottle until used for GC-MS analysis and bioassays.

### 2.2. Gas Chromatography-Mass Spectrometry (GC-MS) Analysis

GC-MS analysis was carried out on 7890 A GC System (Agilent Technologies, Greece) fitted with a nonpolar column: (5%-phenyl)-methylpolysiloxane phase capillary column) with column length of 30 m, column internal diameter 0.25 mm, and the stationary phase coating film thickness of 0.25 *µ*m. Prior to analysis, the essential oil was diluted with *n*-hexane in 10 : 1 ratio. The diluted essential oil (1 *µ*l) was injected at 240°C by using helium as carrier gas at a flow rate of 0.9 ml/min. Then, the gas chromatograph oven temperature was programmed to 60°C for 1 min and set at a rate of 3°C/min until the temperature reached 240°C. The interface temperature was 280°C. When the GC-separated component of the essential oil reached the mass spectrometer (5975C VL MSD, Agilent Technologies, Greece), it was ionized by 70 eV ionization voltage with a source temperature of 230°C. The ionized samples were accelerated and separated according to mass to charge ratio (m/z) by a quadrupole mass analyzer with a temperature of 150°C. Finally, the ionized species were detected by a MS detector with a scanning capacity of 40–400 amu. The compounds were identified by comparing the obtained mass spectra with the mass spectra from the database.

### 2.3. Larvicidal Bioassay

The larvicidal bioassay test of the essential oil was carried out following the standard WHO protocol [15]. A group of fourth instar *An. arabiensis* larvae (*n* = 25) were immersed by using a dropper in white enamel cups that contained water and various concentrations of test samples (6.25, 12.5, 25, 50, 100, 200, and 400 ppm). Then, the cups were kept at a temperature of 27 ± 2°C and relative humidity of 75 ± 10% with a photoperiod of 12 h light and 12 h dark cycles. The experiment was conducted in four replicates for each concentration of test samples and controls. Larval mortalities were recorded after 24 and 48 h of exposure, and then the percentage of larval mortalities was calculated by using the following formula:

(1) $$\begin{array}{c} \text{percentage}\;\text{of}\;\text{larval}\;\text{mortalily}=\frac{\text{number}\;\text{of}\;\text{dead}\;\text{larvae}}{\text{total}\;\text{larvae}\;\text{introduced}}X\;100. \end{array}$$

### 2.4. Ovicidal Activity Test

The essential oil was assayed for its ovicidal activity against freshly laid *An. arabiensis* eggs as per the method described by Elango et al. [16] with minor modifications. The same test concentrations were used as in larvicidal bioassay tests. In brief, *An*. *arabiensis* eggs (*n* = 25) were counted under a stereo zooming Olympus SZ40 microscope (Olympus, Japan) and immersed by using a dropper to white enamel cups that contained different concentrations of the oil. Egg hatchability was assessed with the same microscope after incubation for 72 h. The ovicidal effect was calculated in percentage by dividing the number of hatched eggs by the total number of eggs immersed times 100 as shown in the following equation:

(2) $$\begin{array}{c} \text{percentage}\;\text{of}\;\text{hatchabilily}=\frac{\text{number}\;\text{of}\;\text{hatched}\;\text{eggs}}{\text{total}\;\text{number}\;\text{of}\;\text{eggs}}X100. \end{array}$$

### 2.5. Repellent Activity Test

The repellent activity of the essential oil against adult female *An. arabiensis* was performed following the WHO mosquito repellence test protocol [17]. Fifty nulliparous 3–5 days old female mosquitos which starved for about 12 h were placed into a test cage during all tests in the laboratory. Before the experiment began, three volunteers washed their hands and forearms with scent-free soaps and dried them for 10 min before extracts and control application.

One ml each of 366.7, 133.3, and 41.6 *µ*g/cm^2^ of the essential oil and ethanol (diluent) was applied to approximately 600 cm^2^ of the forearm. The treated and control arms were placed every 30 min in the mosquito cage for 3 min observation period. The periods of observation were 30, 60, 90, 120, 150, 180, 210, and 240 min. Mosquitoes that land/probe on the forearm were recorded. Tests were conducted during the day in the dark room. Each test concentration was repeated three times with three volunteers. Formulated commercial 20% N, N-diethyl-meta-toluamide (DEET) (CAS no. 134-62-3) was used as a standard for comparison of protection time to that of the test repellents. The percentage of protection is calculated for each concentration by using the following formula:

(3) $$\begin{array}{c} \text{percentage}\;\text{of}\;\text{protection}=\frac{C-T}{C}X\;100, \end{array}$$

where C and T are the number of mosquito landings on the forearm of negative control and treated skin, respectively.

### 2.6. Statistical Analyses

The average mortality of larvae and hatchability of eggs data and other statistics were subjected to probit analysis for calculating data such as LC_50_, LC_90_, IH_50_, IH_90_, ED_50_, ED_90_, and ED_99_ at 95% confidence intervals, and Chi-square values were calculated using the SPSS Statistical software package 26 version. After the normality test of data, one-way analysis of variance (ANOVA) was used to determine the level of significance of the effect of different concentrations on the exposed mosquitoes. Results with *p* < 0.05 were considered statistically significant.

## 3. Results and Discussion

### 3.1. Chemical Composition

Hydrodistillation of the dried leaves of *L. stachydiformis* yielded 0.36% (w/w) of a pale, yellow-colored essential oil with a distinct smell. GC-MS analysis identified a total of 24 compounds representing 90.34% of the total oil. The essential oil was found to be rich in sesquiterpene hydrocarbons (45.07%), followed by oxygenated sesquiterpenes (30.27%), oxygenated monoterpenes (7.03%), diterpene hydrocarbons (6.81%), monoterpene hydrocarbons (0.24%), and others (0.92%). As shown in Table 1, the major components of the oil were caryophyllene oxide (19.84%), germacrene D (19.06%), *trans*-caryophyllene (12.29%), phytol (6.81%), *α*-humulene (4.81%), humulene epoxide II (3.57%), linalool (3.33%), and spathulenol (2.77%). Although the major components of the oil were previously isolated from other *Leucas* species, this is the first report on the identification of the monoterpenes, *trans*-Verbenol (1.97%), and verbenone (0.46%) and the sesquiterpene hydrocarbon modheph-2-ene (0.37%) in the genus *Leucas*.

### 3.2. Larvicidal Activity

Plants with insecticidal activity have a significant role in both public health and agriculture. These plants are crucial in controlling disease vectors, providing sustainable alternatives to chemical pesticides, and reducing risks to human health and the environment [18]. The study and use of natural products of plant origin such as extracts and essential oils are fundamental to overcoming the challenges associated with disease-transmitting mosquito vectors and vector-borne diseases [19]. Malaria vector control at larvae stages can be advantageous because the larvae are concentrated in particular habitats, relatively immobile, and occupy minimal habitat areas as compared with adults that can rapidly disperse over large areas [20]. The killing of early-stage mosquitoes will also decrease the future population of mosquitoes that are resistant to insecticides in the environment [21]. Various extracts and essential oils from the Lamiaceae and other families have been known to exhibit mosquito larvicidal activity. For instance, the ethanol extract of the whole plant of *Leucas aspera* exhibited significant larvicidal activity against the first to fourth instar larvae of *An. stephensi* after 24 h exposure [22]. In this study, the essential oil of *L. stachydiformis* exerted concentration-dependent larvicidal activity against the fourth instar *An. arabiensis* larvae (Table 2). At a concentration of 400 ppm, 92% of the larvae were killed within 24 h of exposure and then rose to 96% after 48 h of exposure (Table 2). According to Felipe Oliveros-Díaz et al.'s [23] larvicidal efficiency classification, the essential oil of *L. stachydiformis* showed high larvicidal activity at a concentration of 400 ppm and 200 ppm without significant difference after 24 h and 48 h exposure (*p* > 0.05). However, at concentrations less than 200 ppm, the activity was concentration-dependent (*p* < 0.05). The present results are comparable with the findings of Thomas et al. [24], where a concentration-dependent larvicidal action was observed for the essential oils of *Syzygium aromaticum* (clove) and *Cinnamomum verum* (cinnamon) against *An. gambiae*.

At concentrations of 200 ppm and 100 ppm, the essential oil of *L. stachydiformis* caused 88% and 72% larval mortality in 24 h, respectively. These mortality percentages are slightly less than the larvicidal effect of the essential oils of *Thymus serrulatus* and *Thymus schimperi*, which exerted 100% larval mortality against *Anopheles arabiensis* larvae at 100 ppm and 200 ppm. This potency difference may be due to the qualitative and quantitative variations of essential oil composition between *Thymus* and *Leucas* species [25]. The 88% larval death at 200 ppm of the essential oil of *L. stachydiformis* in 24 h was comparable to the 89.2% *Aedes japonicus* larval death caused by 135 ppm of the essential oil of *H. mantegazzianum* seeds [26]. The mortality rate observed for the negative control after 24 and 48 h of exposure was 0%, and all the larvae were developed into pupae after 48 h.

After 48 h of exposure, the LC_50_ and LC_90_ values of *L. stachydiformis* essential oil were 34.2 ppm and 175.7 ppm, respectively (Table 2).

The 24 h LC_50_ value of the essential oil of *L. stachydiformis* (43.4 ppm) was very similar to that of *L. zeylanica* essential oil (44 ppm) against *Ae. aegypti*. This similarity in potency could be due to the presence of high amounts of caryophyllene oxide, germacrene D, phytol, and *α*-humulene in both essential oils [27]. Moreover, this result is also comparable to the 24 h LC_50_ values of the essential oil of *Hyptis capitata* (39.08 ppm) against *An. stephensi* [28] and *Euclasta condylotricha* (38.46 ppm) against *An. gambiae* [29] and *Pinus sylvestris* essential oil which exhibited significant larvicidal activity against *D. gallinae* larvae with LC_50_ values of 0.68 mg/mL [30].

Larvicidal properties of the essential oil of *L*. *stachydiformis* may be linked to its major constituents such as caryophyllene oxide, germacrene D, linalool, and *α*-humulene which have been reported to have strong larvicidal properties [27, 29–31]. However, the effect of these major constituents might have been strengthened by the cooccurrence of minor compounds that act in an additive and/or synergistic manner [32]. Among the minor constituents of the oil, *α*-pinene, verbenone, bicyclogermacrene, *δ*-cadinene, and *α*-terpineol were reported to have larvicidal activity [1, 29, 32, 33].

Different phytochemicals are known to cause neurotoxicity or direct contact toxicity to the midgut epithelium of mosquito larvae [34]. In the present study, the identified essential oil constituents such as caryophyllene oxide, *α*-pinene, and linalool are known to have antiacetylcholinesterase activity [5], whereas *α*-terpineol was reported to have an influence on the production of cyclic adenosine monophosphate (cAMP) and calcium at the cellular level and these effects consequentially lead to larval death [35].

It was reported that the presence of a variety of compounds in essential oils may lead to a collaborative cell membrane penetration of the mosquito larvae by nonpolar constitutes thereby enhancing the entry of active compounds to the cell, and this effect consequently increases their bioavailability and larvicidal activity [36]. This combination (synergistic) effect may have occurred in the essential oil of *L. stachydiformis* since the main constituents identified are highly lipophilic compounds [37]. It has been reported that combinatorial action does not always lead to a positive effect, i.e., the isolated pure compounds may have a greater larvicidal effect than the essential oil from which the compound is isolated. For example, Govindarajan et al. [38] reported that the larvicidal activity of pure (Z)-*γ*-bisabolene isolated from the essential oil of *Galinsoga parviflora* (Asteraceae) is much higher than that of the oil itself.

### 3.3. Ovicidal Activity

Controlling of egg hatching before larval emergence is another efficient approach for malarial vector management. To withstand unfavorable environmental conditions such as dryness and severe temperatures, mosquito eggs are encapsulated with a chorionic membrane that hardens over time throughout embryonic development. As a result, using ovicides in the early phases of egg development is the best way to reduce mosquito population growth [39].

In the present study, ovicidal activity was performed for the first time against *Anopheles arabiensis* eggs. The mean percentage hatchability of the eggs was observed post 72 h exposure to the essential oil of *L*. *stachydiformis*. It was found that the mean percentage hatchability was inversely proportional to the concentration of the essential oil [37]. The essential oil of *L. stachydiformis* showed mean percentage hatching that ranges from 0% at 400 ppm to 90% at 6.25 ppm (Table 3).

The zero hatchability induced to *An. arabiensis* eggs at 400 ppm of the essential oil agrees with the finding of Govindarajan and Rajeswary [40], *who* reported zero hatchability of *An. stephensi* eggs at 375 ppm for benzene fraction of *Albizia lebbeck* seed extract. Govindarajan et al. [41] also reported that the ethyl acetate leaf extract of *Acalypha indica* exhibited 16.3% of *An. Stephensi* eggs hatchability at 100 ppm. This finding is roughly comparable with the present finding that 25% of *An. arabiensis* eggs were hatched at 100 ppm of the essential oil of *L. stachydiformis*. In the present study, 36% of the eggs were hatched after exposure to 50 ppm of the essential oil, which is comparable to 34.72% of hatching of *An. stephensi* eggs exhibited by 50 ppm of the ethanol extract of *Solena amplexicaulis* [42].

Nonpolar constituents of the essential oil are likely to be responsible for the ovicidal activity because such constituents have been reported to cause neurotoxicity or difficulty in respiration due to blockage of the aeropyles of eggs by the oil layer [43].

The data from probit analysis revealed that more concentrated oil was needed to reach 50% hatching inhibition than killing 50% of the larvae. This could be due to the fact that eggs are encapsulated by a chorionic membrane which makes them more resistant to physical or chemical stress [44].

### 3.4. Repellent Activity

*Anopheles arabiensis* is an opportunistic feeder, showing elasticity in both resting and feeding habits with zoophagic and anthropophagic behavior [45, 46], but it feeds more frequently on cattle and on unprotected humans outdoors (exophagy) where protections are less likely [47]. The use of a bed net may be ineffective if there is a trend toward early evening mosquito bites and exophagic (outdoor) feeding habits like those of *An. arabiensis*. As such, the use of repellents as a method of preventing malaria is particularly promising [48]. Essential oils and extracts of plants are emerging as potential agents for *Anopheles* spp. control [49].

**Figure 1:**
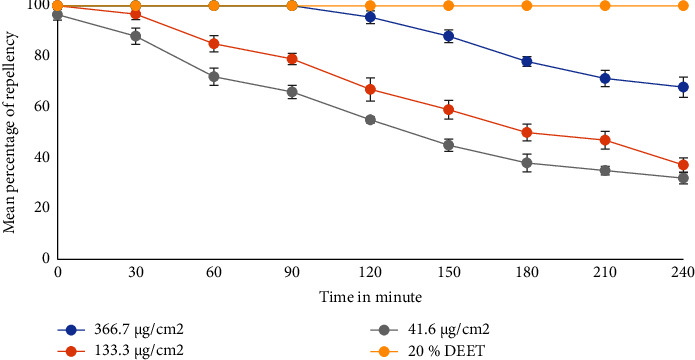
Mean percentage repellent activity of the essential oil of the leaves of *Leucas stachydiformis* against *Anopheles arabiensis* at different concentrations.

As shown in Figure 1, the mean percentage of repellency (MPR) of the essential oil at different concentrations in ethanol was compared with 20% DEET in 30 min intervals until 240 min. The oil gave protection against mosquito bites without any allergic reaction to the test persons. The total percentage protection of the oil for 4 h was 67.9 ± 4.2% at 366.7 *µ*g/cm^2^, 37.2 ± 2.8% at 133.3 *µ*g/cm^2^, and 32 ± 2.2% at 41.6 *µ*g/cm^2^. The 96.7 ± 2.2% MPR of 8% (133.3 *µ*g/cm^2^) of the oil in 30 min was almost equivalent to 1000 ppm (0.1%) of the essential oil of *Millettia ferruginea* which gave 97.12 ± 1.67% protection against *An. arabiensis* in 20 min [50]. Similarly, the 37.2 ± 2.8% MPR of this concentration in 4 h was also comparable to the methanolic leaf extract of *Tribulus terrestris*, which showed 39.2% at 1.0 mg/cm^2^ in 4 h exposure [51]. Furthermore, the 4 h 67.9 ± 4.2% MPR of 22% (366.7 *µ*g/cm^2^) was comparable to 3 h 71% MPR of 20% *Azadirachta indica* oil against field *An. arabiensis* [52].

Among the tested concentrations, the highest MPR was recorded for the 22% (366.7 *µ*g/cm^2^) oil which provided a percentage repellency of 100% in the first 90 min (complete protection time (CPR)), but gradually decreased to 67.9 ± 4.2% after 240 min (4 h). The 4 h MPR of this concentration was comparable to 20% *Cymbopogon nardus* that generated protection of around 70% at 4 h observation against *An. Arabiensis* [53].

According to Dugassa et al. [54], *An. arabiensis* has peaks occurring in both the first and second half of the night. Therefore, the essential oil of *L. stachydiformis* may not be suitable for use for the whole night because of its short CPT (90 min). However, it can be applied in the early evening, i.e., before bedtime to reduce the early biting of mosquitoes, and the late indoor biting can be prevented by mosquito nets.

Repellent properties of the essential oil of *L. stachydiformis* may be associated with the presence of monoterpenoids and sesquiterpenes [55]. The major constituents of the oil, i.e., caryophyllene oxide and germacrene D have been reported to have strong repellent activities [44, 56]. According to Shoukat et al. [57], these compounds are less volatile and have a long-lasting effect as a repellent. Similarly, among the minor constituents of the oil, *α*-terpineol [58], linalool [59], *α*-pinene [60], and *δ*-cadinene [61] have demonstrated mosquito repellent effects. Therefore, the mosquito-repellent effect of *L. stachydiformis* oil against *An. arabiensis* is in full or in part attributed to the abovementioned compounds.

Generally, phytochemicals do also have an advantage since their toxic effects on nonnontarget organisms are less, and they are biodegradable and disappear from the environment within a short period of time [62], whereas synthetic larvicides can continuously contaminate the water environment and are toxic to nontarget organisms [37, 63].

## 4. Conclusion

The present study revealed that the essential oil extracted by hydrodistillation of the leaves of *L. stachydiformis* was rich in sesquiterpenes hydrocarbons and oxygenated sesquiterpenes such as caryophyllene oxide, germacrene D, *trans*-caryophyllene, linalool, and phytol. Also, the essential oil demonstrated potent larvicidal, ovicidal, and repellent activities against *An. arabiensis*. Thus, the current study provided evidence that the leaves of *L. stachydiformis* have the potential for the development of natural insecticides for the control of mosquitoes.

## Figures and Tables

**Table 1:** Chemical compositions of the essential oil isolated from the leaves of *Leuca*s *stachydiformis*.

No.	Components	RT^a^	RI (HP5-MS)^b^	Area or content (%)
1	*α*-Pinene	5.19	931	0.24
2	1-Octen-3-ol	6.37	977	0.92
3	Linalool	10.48	1100	3.33
4	*trans*-Verbenol	12.21	1142	1.97
5	*α*-Terpineol	14.10	1189	1.26
6	Verbenone	14.84	1207	0.46
7	Modheph-2-ene	21.93	1376	0.37
8	*β*-Bourbonene	22.18	1382	1.49
9	*trans*-caryophyllene	23.57	1415	12.29
10	*α*-Humulene	24.94	1450	4.81
11	*γ*-Muurolene	25.92	1474	2.00
12	Germacrene D	26.07	1478	19.06
13	*trans*-*β*-ionone	26.31	1483	0.55
14	Bicyclogermacrene	26.68	1493	1.62
15	*δ*-Cadinene	27.78	1521	0.54
16	Germacrene B	28.99	1552	2.35
17	Spathulenol	29.79	1573	2.77
18	Caryophyllene oxide	29.98	1578	19.84
19	Humulene epoxide II	30.96	1604	3.57
20	*τ*-Cadinol	32.20	1638	1.14
21	*α*-Cadinol	32.69	1651	1.55
22	15-Hydroxy-*α*-muurolene	33.81	1682	0.38
23	6,10,14-Trimethyl-2-pentadecanone	39.47	1844	1.04
24	Phytol	47.90	2111	6.81
	Sesquiterpene hydrocarbons			45.07
	Oxygenated sesquiterpenes			30.27
	Oxygenated monoterpene			7.03
	Hydrogenated diterpene			6.81
	Monoterpene hydrocarbons			0.24
	Others			0.92
	Total identified			90.34

^a^RT, retention time; ^b^RI, Kovats retention indices determined relative to a series of *n*-alkanes (C9–C29) on a nonpolar (HP5-MS capillary) column.

**Table 2:** Larvicidal activity of the essential oil of *Leucas stachydiformis* against fourth instar *Anopheles arabiensis* larvae.

Concentration (ppm)	Mortality % ± SD			LC_50_ (95% CI) ppm	LC_90_ (95% CI) ppm	Chi-square
	24 h	48 h				

400	92 ± 0.5	96 ± 0.7		43.4	229.7	6.3
200	88 ± 1.2	92 ± 1.5	24 h	(31.8–58.5)	(151.6–430.9)	
100	72 ± 0.9	72 ± 1.3				
50	56 ± 1.3	68 ± 1.1				
25	52 ± 2.5	60 ± 1.8		34.2	175.7	8.3
12.5	8 ± 0.6	8 ± 1.6	48 h	(25.0–45.7)	(118.3–317.9)	
6.25	4 ± 0.2	8 ± 0.5				
Negative control	0 ± 0.0	0 ± 0.0				

Values were based on seven concentrations and four replications with 25 larvae in each. Chi-square values were significant at *p* < 0.05 level. LC_50_, 50% lethal concentration; LC_90_, 90% lethal concentration; CI, confidence interval; negative control, 2% dimethyl sulfoxide (DMSO).

**Table 3:** Hatchability of *Anopheles arabiensis* eggs after 72 h of exposure with essential oil of *Leucas stachydiformis*.

Concentration (ppm)	Hatchability % ± SD	IH_50_ (95% CI) ppm	IH_90_ (95% CI) ppm	Chi-square
400	0 ± 0.0	32.2 (24.1–42.5)	144.7 (99.8–252.3)	4.1
200	16 ± 2.2			
100	25 ± 3.5			
50	36 ± 1.4			
25	50 ± 1.2			
12.5	85 ± 2.5			
6.25	90 ± 1.5			
Negative control	100 ± 0.0			

Values were based on seven concentrations and four replications with 25 eggs in each test cup. Chi-square value was significant at *p* < 0.05 level. IH_50_, 50% hatching inhibition concentration; IH_90_, 90% hatching inhibition concentration; CI, confidence interval.

## Data Availability

The data used to support the findings of the study are available from the corresponding author upon request.
